# Evolution of the humanitarian roster system for sexual and reproductive health

**DOI:** 10.1080/26410397.2026.2686033

**Published:** 2026-06-26

**Authors:** Alison Greer, Rima Pai, Mihoko Tanabe, Lara Martin, Nguyen Toan Tran

**Affiliations:** a Senior Advisor, Women's Refugee Commission, New York, NY, USA.; b Doctoral Student, Hubert Department of Global Health Center for Humanitarian Emergencies, Emory University, Rollins School of Public Health, Atlanta, USA; c Assistant Professor, Department of Family Medicine, Boston University Chobanian & Avedisian School of Medicine, Boston, USA; d Manager, Education and Programs, Hubert Department of Global Health Center for Humanitarian Emergencies, Rollins School of Public Health Atlanta, Emory University, Atlanta, GA, USA; e Consultant, Women's Refugee Commission, New York, NY, USA; Adjunct Professor, Faculty of Health, Australian Centre for Public and Population Health Research, University of Technology Sydney, Sydney, NSW, Australia; Privat Docent, Faculty of Medicine, University of Geneva, Genève, Switzerland

**Keywords:** rosters, roster management, humanitarian crisis, surge capacity, humanitarian surge, emergencies, human resources, sexual and reproductive health

## Abstract

Evidence suggests that the number, scale, and severity of emergencies exceed the international humanitarian system's existing capacity and available resources to meet humanitarian needs, especially for the health sector of which sexual and reproductive health (SRH) is an essential component. Since 1997, the Inter-Agency Working Group on Reproductive Health in Crises's Minimum Initial Service Package (MISP) for SRH has been the standard of care for SRH interventions in emergencies, aimed to protect the health, dignity, and rights of women and girls living in humanitarian emergencies. Humanitarian response organisations, national governments, and donors increasingly recognise the importance of MISP implementation at the onset of an emergency. However, an effective humanitarian response demands rapid mobilisation of resources, including the deployment of trained professionals capable of navigating complex scenarios. Roster systems are a human resource tool that maintains a diverse range of qualified professional profiles for emergency preparedness, response, and recovery efforts. Recent trends indicate a shift from a global to an increasingly geographic-specific roster management and recruitment model, which we argue is vital in localising and sustaining humanitarian response. Organisations should channel investments into further strengthening national and regional response capacities, including community-level readiness for SRH, and reconsider international standby roster models. As first responders, communities are best positioned to initiate community-based responses when integrated into all levels of health disaster risk management systems. Fostering robust partnerships between international, regional, national, sub-national, and community levels is critical to ensuring preparedness and response efforts for SRH, resilient recovery strategies, and building back better.

## Résumé

Il apparaît que le nombre, l’ampleur et la gravité des crises dépassent les capacités et les ressources dont dispose le système humanitaire international pour répondre aux besoins humanitaires, spécialement dans le secteur de la santé dont la santé sexuelle et reproductive (SSR) est une composante essentielle. Depuis 1997, le dispositif minimum d’urgence (DMU) du Groupe inter-organisations sur la santé reproductive en situations de crise humanitaire constitue la norme pour les interventions de SSR dans les situations d’urgence visant à protéger la santé, la dignité et les droits des femmes et des filles vivant dans des contextes de crise humanitaire. Les organisations humanitaires, les gouvernements nationaux et les donateurs reconnaissent de plus en plus l’importance de la mise en œuvre du DMU dès le début d’une crise. Néanmoins, une intervention humanitaire efficace exige une mobilisation rapide des ressources, notamment le déploiement de professionnels formés, capables de faire face à des situations complexes. Les systèmes de fichiers de candidats sont un outil de gestion des ressources humaines permettant de maintenir un large éventail de profils professionnels qualifiés pour la préparation, la réponse et le relèvement en cas d’urgence. Les récentes tendances indiquent une évolution d’un modèle de gestion et de recrutement du personnel à l’échelle mondiale vers un modèle plus ancré dans des lieux géographiques spécifiques, ce qui nous semble nous semble essentiel pour localiser et pérenniser la réponse humanitaire. Les organisations devraient investir pour renforcer les capacités d’intervention nationales et régionales, notamment en matière de préparation communautaire pour la SSR, et revoir les modèles de fichiers internationaux de candidats. En qualité de premières intervenantes, les communautés sont les mieux placées pour lancer les interventions communautaires quand elles sont intégrées à tous les niveaux des systèmes de gestion des risques de catastrophe sanitaire. Il est essentiel de favoriser de solides partenariats entre les niveaux international, régional, national, sous-national et communautaire pour garantir la préparation et les interventions en matière de SSR, pour appliquer des stratégies de relèvement résilientes et pour reconstruire en mieux.

## Resumen

La evidencia indica que el número, la escala y la gravedad de emergencias sobrepasan la capacidad actual y los recursos disponibles del sistema humanitario internacional para atender las necesidades humanitarias, especialmente en el sector salud del cual la salud sexual y reproductiva (SSR) es un componente esencial. Desde 1997, el Paquete de Servicios Iniciales Mínimos (PSIM) del Grupo de Trabajo Interinstitucional sobre Salud Reproductiva en Situaciones de Crisis para SSR ha sido el estándar de atención para intervenciones de SSR en emergencias, con el objetivo de proteger la salud, la dignidad y los derechos de las mujeres y niñas que viven en situaciones de emergencias humanitarias. Cada vez más, las organizaciones de respuesta humanitaria, gobiernos nacionales y donantes reconocen la importancia de la aplicación del PSIM al inicio de una emergencia. Sin embargo, una respuesta humanitaria eficaz exige la movilización rápida de recursos, que implica el despliegue de profesionales calificados capaces de navegar escenarios complejos. Los sistemas de turnos son una herramienta de recursos humanos que mantiene una gama diversa de perfiles de profesionales calificados para la preparación, respuesta y esfuerzos de recuperación ante emergencias. Las tendencias recientes indican un giro del modelo de administración de turnos y reclutamiento mundial a un modelo cada vez más específico según la región geográfica, lo que argumentamos es vital para adaptar al entorno y sustentar la respuesta humanitaria. Las organizaciones deben canalizar las inversiones hacia continuar reforzando la capacidad de respuesta a nivel nacional y regional, que implica la preparación a nivel comunitario para SSR, y reconsiderar modelos de turnos internacionales en espera. Como socorristas, las comunidades se encuentran en la mejor posición para iniciar respuestas comunitarias cuando son integradas en todos los niveles de los sistemas de gestión de riesgos de desastres en salud. Fomentar alianzas robustas entre los niveles internacional, regional, nacional, subnacional y comunitario es fundamental para garantizar la preparación y los esfuerzos de respuesta para SSR, estrategias de recuperación resilientes y reconstruir mejor.

## Introduction

Sexual and reproductive health (SRH) is an essential component of the human right to health and lifesaving health programs and services in humanitarian settings.^1–3^ However, evidence suggests that the number, scale, and severity of emergencies exceed the international humanitarian system's existing capacity and available resources to meet the wide range of health – including SRH – needs that typically accompany rapid-onset emergencies.^4,5^ An effective humanitarian response demands timely mobilisation of resources, including deployments of trained professionals to respond appropriately to complex situations.^6^ Local and national capacity can become overwhelmed in an emergency, leading to demand for international deployments of humanitarian and other specialists.^7^ Surge rosters are one well-known approach to rapidly meet emerging critical human resource needs at sub-national, national, regional, and international levels, to ensure essential services remain available during humanitarian emergencies.^8^

Decades of research have shown that women and girls living in humanitarian contexts experience increased risks and incidence of sexual violence, transmission of and health complications due to untreated or inadequately managed HIV and other sexually transmitted infections, maternal and newborn death or injury, unintended pregnancies, and unsafe abortions.^3,9–16^ Aiming to save the lives and protect the health, dignity, and rights of women and girls living in crisis affected settings, the Inter-Agency Working Group on Reproductive Health in Crises – an international coalition of organisational and individual members committed to advancing SRH and rights in humanitarian and fragile contexts – developed the Minimum Initial Service Package (MISP) for Sexual and Reproductive Health. The MISP has been the international standard of care for SRH interventions in emergencies since 1996, with revisions made to its flagship *Inter-Agency Field Manual for Reproductive Health in Humanitarian Settings* in 1999, 2010, and 2018.^16–19^

The first MISP objective calls for the Health Sector or Cluster to identify an organisation to lead on MISP implementation.^16^ Humanitarian response organisations, national governments, and donors increasingly recognised and documented the importance of implementing the MISP at an emergency’s onset and championed the integration of SRH within evolving humanitarian standards, practices, and coordination architecture.^20^ In 2005, the cluster system was established as part of a humanitarian reform process to improve the delivery of lifesaving services during an emergency response by strengthening global – and when activated, national or regional – humanitarian coordination, accountability, and effectiveness within and across service delivery areas.^21^ Currently, there are 11 global clusters for key technical areas, including the Global Health Cluster under which the SRH Task Team was established in 2022.^22,23^ Such developments aim to standardise and strengthen SRH coordination at the highest levels of humanitarian response for activation on the ground.^17,23^ Additionally, they reinforced the recognition of SRH within the Protection Cluster around gender-based violence, and the Logistics Cluster where it informs medical logistics operations.^16,23^

This paper describes the evolution of humanitarian surge rosters, especially for SRH services, in the context of global movements to localise humanitarian response through strengthening regional, national, and local capacities.

## International humanitarian surge capacity and rosters: definitions and context

An international humanitarian response is activated when national or regional capacity to respond is overwhelmed and unable to ensure the needs and human rights of crisis-affected persons.^24,25^ Rosters are human resource mechanisms used to quickly identify and deploy qualified national, regional, and international emergency responders. They are strategic, managed compilations of professionals with specific and diverse profiles whose qualifications, suitability, and availability are assessed for rapid deployment.^6,26^ The three-year-long Transforming Surge Capacity Project was launched by the Start Network – a network of non-governmental organisations (NGOs) from around the world committed to making fundamental changes within the humanitarian system – to promote civil society participation in humanitarian surge capacity.^27^ Surge capacity, as defined by the Project, is “The ability of organisations, communities, and individuals in crisis to rapidly and effectively respond to the needs of affected populations through improved local preparedness, collaborative effort, and the scaling up and down of responses”.^28^

Within the health sector, global networks and organisations such as the International Federation of Red Cross and Red Crescent Societies (IFRC) developed surge capacity in the form of Emergency Medical Teams and Units that can include a public health response.^29^ National governments have deployable response teams, such as the United States Centers for Disease Control and Prevention’s (CDC) Global Rapid Response Team.^30^ Agencies have also implemented shared roster systems. One example is the Standby Partnership Programme mechanism that comprises 16 United Nations (UN) agencies – including the United Nations Population Fund (UNFPA), 56 Standby Partners that include government agencies, NGOs, and businesses, and 400 roster members operating under an agreed Memorandum of Understanding (MOU) in response to a humanitarian emergency.^31^ Training programs have been created to standardise Emergency Medical Teams and enhance collaboration and teamwork.^32,33^

For SRH, UNFPA operates the Global Emergency (Surge) Roster to ensure SRH services in crisis, recovery, and post-crisis situations. Members are expected to be deployable within 48–72 hours' notice. Members’ tasks include facilitating procurement and delivery of emergency medical supplies and equipment while spearheading coordination and implementation of the MISP alongside activities implemented by national and international actors.^34^ In 2018, based on available data, UNFPA deployed 16 internal staff, 32 external staff (retirees, previous staff, consultants, or other qualified persons), and 41 Standby Partners. Of these deployments, 21% were specifically for SRH coordination, while 52% focused on gender-based violence coordination.^35^ During the Covid-19 pandemic, UNFPA adapted its global Surge to be responsive to travel restrictions and evolving needs by deploying remote and local Surge members. In 2020, there were 92 deployed members, a number which steadily increased over the next couple of years to 150 deployed in 2023 – comparable to the 149 Standby Partner deployments by UNHCR.^36,37^ This increase in deployments was facilitated by updated recruitment processes with new guidelines and vetting processes to reach a wider pool of candidates, and use of feedback mechanisms and assessment tools. Impacted by devastating funding cuts to SRH and rights development and humanitarian funding by critical donors over the past couple of years, in 2025 the number of deployed UNFPA Surge members decreased to 75.^37,38^

## Challenges to humanitarian roster systems

Although rosters are a commonly used human resource practice in humanitarian contexts, a range of budgetary, logistical, administrative, human resource, and other inherent challenges persist in maintaining effective and flexible systems. Emergency response funds vary in their ability to respond rapidly and support capacity strengthening activities, especially amidst the backdrop of well-documented, prevailing coordination challenges in humanitarian response, and data sharing difficulties between organisations at large.^39,40^

IFRC’s review of their Emergency Medical Team and Unit deployments from 2015 to 2019 identified areas for improvement, including needs assessments, modularity, context-appropriate support and integration, human resources, monitoring and evaluation, and adapting responses to diverse emergencies. The evaluation’s authors stress a greater need for focus on improving public health response over clinical surge responses, standardising deployment criteria and standard operating procedures (SOPs), and strengthening regional and national capacity to facilitate higher quality, sustainable, and more cost-effective emergency responses. They further underscore the need for flexibility and longer-term health programming – distinct from health surge responses – that focuses on health systems strengthening and more effective transitions from emergency to recovery and longer-term programming, particularly where crises become protracted.^29^ Similarly, the CDC’s review of their Global Rapid Response Team deployments across 21 countries between 2016 and 2018 recognised challenges in identifying, selecting, and ensuring the availability of deployable and effectively trained surge staff in their roster’s development and maintenance. They concluded that the lack of defined SOPs covering both non-emergency maintenance measures and multi-stage emergency response processes delayed response times and effectiveness in some deployments.^41^ Additionally, interviews with emergency responders deployed to Cox’s Bazar, Bangladesh for the Rohingya response by the World Health Organisation through operational partnerships, raised challenges and opportunities-related themes of staffing, deployments, the physical and operational office environment, and capacity strengthening. Professionals interviewed noted the need for longer-term staffing plans once the situation is determined to be a protracted humanitarian response, citing issues around heavy workloads, work continuity amidst short-term staff rotations, the effectiveness of existing performance review processes, insufficient professional and well-being support, and clarity of terms of reference. Interviewees discussed the importance of including more local organisations in the partnership and recruiting more professionals with specific technical backgrounds as well as those local to the context with the necessary language and cultural expertise for operational effectiveness.^42^

Similar challenges have been found to impact surge capacity initiatives for SRH. A 2019 evaluation by UNFPA found mixed evidence around the effectiveness of surge and roving team deployments and an overreliance on the mechanism to fill human resource gaps. Effectiveness depended on the experience, interpersonal skills, and attitude of deployed personnel; support provided by country-level senior management and existing staff; ability of surge deployees to remain dedicated to their specific role; and contract modality which included various internal contracts, consulting or temporary appointment contracts, or Standby Partner contracts.^35^ More recently, UNFPA’s 2025 evaluation of its humanitarian capacity cites the costliness of its Surge mechanism as well as its value as a rapid, reliable mechanism to supplement humanitarian SRH expertise when needed. The evaluation notes challenges at the country level with hiring humanitarian staff due to long recruitment and hiring processes done at the central level and unpredictable and short-term funding which results in high staff turnover. Additionally, existing organisational Surge recruitment processes and procedures do not facilitate local roster member recruitment, although some Country Offices reported fostering networks of national experts that can be called upon in emergencies.^37^ The Transforming Surge Project recommends that roster member profiles be accessible to organisations with interconnected databases spanning from sub-national to international levels to expeditiously identify suitable candidates and facilitate their deployments.^28^

## Evolving roster systems

Current trends demonstrate shifts from **global** to **increasingly geographic-specific roster management and recruitment models**.^28,43^ Alternative human resource methodologies and mechanisms are being explored to widen recruitment opportunities beyond traditional approaches, both internally and externally, depending on organisational structures and geographic foci. For example, UNFPA is developing regional emergency surge rosters with geographic-specific knowledge and experience. The first launched in the Pacific Region in 2023 and includes specialists from within and beyond UNFPA.^44^ Another is planned for West and Central Africa and will consist of internal UNFPA regional Francophone SRH and GBV experts to strengthen the region’s emergency preparedness and response.^45^

### Localising humanitarian response

At the 2016 World Humanitarian Summit, commitments were made by donors, UN agencies, international and national NGOs, and IFRC in the *Grand Bargain* and its focus on strengthening regional, national, and especially local responses to emergencies. These commitments spearheaded new ways for actors to respond to humanitarian crises and decouple humanitarian practices from colonial legacies, power relations, and ideology.^46^ To honour pledges to allocate at least 25% of humanitarian funding to local and national responders who traditionally received less than 1% of support, organisations are shifting from international standby roster models and **investing in strengthening national and regional capacity**.^47^ These localisation endeavours seek to leverage the knowledge, skills, and experiences of responders from crisis-affected and fragile settings.^48,49^ Almost a decade later, in 2025, the Inter-Agency Standing Committee, the foremost humanitarian coordination mechanism globally, pledged to build on and operationalise the humanitarian reforms prioritised in the *Grand Bargain* with renewed urgency through the Humanitarian Reset.^25,50^

In 2018, the Transforming Surge Capacity Project published a report entitled *The Future of Humanitarian Surge*, focusing on eight themes: localised approaches to surge, collaborative surge practices, engagement with the wider sector, understanding surge-related capacity strengthening needs and gaps, sustainability, the role of women in the surge, well-being of surge responders, and sharing of best practices in human resource management. According to the Project, localised surge initiatives enhance the ability to identify needs, improve contextual understanding, expedite response times, and ensure cost-effectiveness. Collaborative partnerships, alongside tools like joint rosters, shared services, coordination mechanisms, and preparedness protocols, can augment the societal impact of local and national actors.^28^

In view of prioritising training at the onset of an emergency, learnings from IFRC responses emphasise that strengthening national and regional capacities – both in preparedness and response phases – is critical to enhancing health surge response. The review’s authors argue that early investments in public health and preventative measures are scalable, comparatively cost-effective, and vital in preventing post-emergency declines in population health. They especially note that such investments will be critical as health surge responses will undoubtedly be led by local and regional actors, requiring international organisations traditionally engaged in clinical surge responses to better leverage and coordinate with national and regional actors for a locally-led response.^29^

The SRH in Crisis and Post-crisis Situations (SPRINT) Initiative’s review of the SRH response in Fiji in 2016 and Tonga in 2018 underscores the benefits of national capacity strengthening investments and creation of coordination structures before an emergency. The review found that the existing training program in Tonga, combined with the International Planned Parenthood Federation (IPPF) Humanitarian Hub investments, and Tonga Family Health Association’s prominent role in delivering SRH services, experience with contract work implementation under short timeframes, and strong relationships with the MoH, facilitated a relatively smooth and rapid response to tropical cyclones Winston and Gita. In contrast, limited prior capacity strengthening efforts in Fiji necessitated that trainings take place during the immediate cyclone response with the support of two IPPF surge staff, while unclear stakeholder roles and coordination hampered the response. Despite initial confusion, with the surge staff’s support, trainees in Fiji established a Family Health Sub-cluster to facilitate an SRH response in collaboration with the MoH, medical services teams, and partner organisations. Key informants emphasised the importance of this development, noting that, “Otherwise, reproductive health would have been lost in the health cluster because they had so many other concerns”. The review highlighted the need for comprehensive activities at multiple levels, nationally and regionally, to strengthen SRH response capacity, and the role that surge support can play where needed. The article further emphasised the significance of formalising partnerships, conducting regular meetings, and organising training sessions to “ensure the currency of coordination efforts in readiness for activation”.^51^

Organisations, including UNFPA, are investing in training and partnerships with national and sub-national entities, such as Ministries of Health (MoH) and in-country NGOs, to enhance regional and national capacity and collaboration with sub-national and international NGOs, the private sector, and UN agencies. UNFPA West and Central Africa (WCA) Regional Office, for example, is collaborating with Ministries of Health and Education, professional associations, and other partners in the region to reinforce national emergency SRH capacity and grow a national roster of SRH and GBV experts through integrating the MISP into national midwifery and social protection curricula.^45,52^ UNFPA’s Humanitarian Midwife Program deploys midwives to emergency responses who have received further training in humanitarian preparedness and response and the MISP.^45^ In the field of roster management, there is a growing interest in developing regional platforms for sharing promising practices and resources, accessing learning opportunities, and maintaining shared rosters. Mentoring and buddy systems have been proposed for on-the-job training to strengthen surge personnel’s capacity, along with discussions on transferring relevant skills to national or local staff earlier if external surge support is introduced.^28,35^

Localisation efforts require greater facilitation by international NGOs, while promoting sub-national and national NGOs to assume leadership roles and sustain coordination with authorities and with other national and sub-national actors. Examples of facilitation include seconding staff to provide technical expertise and capacity strengthening; promoting standardised approaches to surge around training and human resources practices; and enabling access to funding for national or sub-national NGOs.

### Community-level disaster risk management

To further disaster risk management at community levels, national actors take a leadership role spearheading national-level responses and are supported by global and regional actors. This involves strengthening linkages with local and sub-national government structures and training with local partners to enhance their ability to implement effective surge responses. This approach would require conditional surge funding to international NGOs and UN agencies contingent on collaboration with local responders, including community first responders who may not traditionally be considered as humanitarian actors.^28^ The *Sendai Framework for Disaster Risk Reduction 2015–2030* emphasises resilience-building and identifies health – specifically SRH – as a critical aspect of strengthening individual and community resilience.^53^ Operationalising the *Sendai Framework* to strengthen community resilience demonstrates that government-led engagement with communities to identify and leverage existing capacities for mitigating risks and vulnerabilities enhances the effectiveness of emergency preparedness activities.^54^

Several initiatives have engaged communities to prepare them to better respond to emergencies. In Pakistan, for example, the Rahnuma-Family Planning Association of Pakistan, CDC, Women’s Refugee Commission, and SPRINT Initiative collaborated to strengthen community capacity to prepare for and respond to SRH risks in selected disaster-prone areas. The project took place in three districts in Khyber-Pakhtunkhwa, Sindh, and Punjab provinces from 2016 to 2020. This initiative began with a MISP training for critical community actors, followed by the development of action plans to strengthen SRH response capacity over one and a half years. Project learning highlighted the benefits of investing in preparedness to improve core SRH services and linking communities to existing formal structures at the national, sub-national, and district levels. Action planning led to immediate gains and longer-term benefits, including the integration of the MISP into disaster risk management efforts at national, sub-national, and district levels. Community mobilisation, awareness-raising, and the creation of blood donor groups and emergency transport contributed to averting mortality at the community level.^55^

Integrating the MISP into pre-service education and curricula for midwives, nurses, medical students, and public health students can also be effective approaches to nurturing future capacity, as demonstrated in Sri Lanka, Fiji, Samoa and the Democratic Republic of Congo.^52,56^ All of these endeavours are crucial to fulfilling *Grand Bargain* commitments, advancing localisation efforts, and responding effectively to humanitarian crises. However, the SPRINT Initiative emphasises the need to institutionalise SRH in emergencies within national policy and accountability mechanisms to ensure financial and political support for community-based initiatives.^51^ Learning from decades of work, the SPRINT Initiative and other organisations underscore the imperative of integrating community-based responses into all tiers of the health system’s disaster risk management framework before emergencies arise.^51,54,55^

Funding for localisation can be challenging to secure. Local organisations often have smaller budgets that may not cover staff relocation, extended deployments, coordination, data sharing and monitoring frameworks, and the overall flexibility that are needed for successful surge capacity. Improved response rates have been reported through investments in country-level emergency preparedness efforts, partnerships with national and sub-national NGOs, and regionalisation of roster systems.^5^ The Start Fund is one example of a financing mechanism that provides anticipatory and rapid response funding to support local actors to anticipate and proactively mitigate the risks of a crisis. Drawing on community priorities, this mechanism has supported local coordination hubs in over 70 countries experiencing cyclic natural disasters and predictable political violence.^57^ Much more research is needed in this area to document efforts around localisation and its impact on SRH humanitarian response capacities.

## The way forward

A review of the evolution of humanitarian roster and surge systems in the context of SRH is not only important for ensuring and sustaining lifesaving SRH services and upholding human rights during and after a humanitarian emergency, it also highlights possibilities for strengthening preparedness and response efforts and resilient recovery strategies. As international humanitarian and development programming and services, especially for SRH and rights, are being defunded and progress is being rolled back, all aspects of a humanitarian response should be assessed to understand where opportunities exist to promote priorities and safeguard the long-term sustainability of services.^58–60^ Leveraging sustainable and equitable collaboration between sub-national, national, regional, and international stakeholders – including Government agencies, UN agencies, NGOs, and others – is particularly crucial for organisations striving for effective, efficient, and impactful roster management in a context of shrinking resources and growing humanitarian needs.^43,61,62^

Over the years, an important shift in support of localisation efforts has occurred – including in surge roster models – presenting opportunities to optimise inter-agency collaborations and advance investments in strengthening and empowering sustainable and coordinated emergency responses at sub-national, national, and regional levels. Recent trends indicate a geo-shifting and decentralisation of rosters, which we argue plays a vital role in localising and sustaining humanitarian response.^28,43^ Equally important is supporting regional and national recruitment of personnel, alongside the cultivation and facilitation of SRH coordination, data sharing, and technical skills for roster deployments.^40^ When effectively executed, such deployments can offer comparative advantage by localising SRH coordination structures. In settings where national or sub-national SRH coordination structures already exist due to preparedness efforts, regional or national surge teams can provide supplementary support as needed. Conversely, in the absence of pre-existing structures, these teams may be tasked with advocating for SRH coordination and MISP prioritisation.

Donor support for surge rosters is critical to sustaining SRH capacity and prioritisation, particularly at regional and national levels. Such support should be restored and grant access to direct funding for regional and national actors to build their surge capacity. Additionally, it should facilitate community readiness for SRH and coordination mechanisms.

For global actors, we recommend direct available support toward regional and national surge services by leveraging donor connections to mobilise and channel resources. This would include promoting common approaches to SRH surge operations, particularly in training and human resources practices, and providing technical expertise such as monitoring, evaluation, reporting, and sectoral guidance through staff secondments. Furthermore, capacity strengthening in disaster risk reduction and emergency response should be provided to regional, national, and community-based actors. For the Health Cluster specifically, we advocate for international support for its localisation strategy, released in June 2024, which will assist with operationalising the localisation priority of its five-year strategic plan. This strategy defines key actions to enhance active participation of and equitable partnership with national and sub-national actors in country Health Clusters and the Global Health Cluster, including in coordination and leadership functions, and opportunities for national and sub-national actors to access more resources. Capacity strengthening and sharing principles and practices play a critical role in this strategy and are important to making greater progress towards locally led emergency health responses, including by facilitating links between United Nations and community-led efforts and resources.^63^ Activities are underway within the SRH Task Team to further systematise and strengthen international, regional, and national SRH coordination capacity in alignment with localisation priorities, including through the development of an SRH Coordination Training that can be widely implemented once available.^23,64^

Regional actors should facilitate and support locally led SRH surge responses, coordinate regional responses, serve as a link between global and national levels, create spaces for national and local representation, and offer capacity strengthening opportunities for national and community-based actors. National actors are recommended to lead national responses with complementary assistance from the global and regional entities, link with local government structures, facilitate sub-national and community-level representation in SRH coordination structures, integrate the MISP into pre-service health professional training, and train community-based organisations in SRH preparedness and response. Sub-national actors should bridge national and community levels, coordinate with other sub-national actors, lead local coordination, create spaces for community representation, and facilitate community readiness for SRH.

Community actors should continue to serve as first responders with support from those at national and sub-national levels, raise community voices with SRH coordination platforms, and regularly reassess SRH preparedness to address gaps in MISP implementation, thereby improving future responses. As first responders, communities are best positioned to initiate localised responses when integrated into all levels of health disaster risk management systems. Moving forward, fostering robust partnerships between international, regional, national, sub-national, and community actors is paramount to ensuring preparedness and response efforts contribute to “building back better” in the aftermath of crises.
